# Comparison of Ultrasound Versus Ultrasound With Nerve Stimulation-Guided Obturator Nerve Block to Prevent Adductor Spasm in Patients Undergoing Transurethral Resection of Bladder Tumor: A Randomized Controlled Study

**DOI:** 10.7759/cureus.53062

**Published:** 2024-01-27

**Authors:** Simmi Muwal, Dharam Singh Meena, Arushi Gupta

**Affiliations:** 1 Anesthesiology and Critical Care, Vardhman Mahavir Medical College and Safdarjung Hospital, New Delhi, IND; 2 Anesthesiology, Vardhman Mahavir Medical College and Safdarjung Hospital, New Delhi, IND

**Keywords:** adductor spasm, onb, turbt, ultrasound, obturator nerve

## Abstract

Background

This study aimed to compare ultrasound versus ultrasound with nerve stimulation-guided obturator nerve block (ONB) for the prevention of adductor spasm in patients undergoing transurethral resection of bladder tumor (TURBT).

Methodology

This randomized controlled study included 240 adult patients in the age group of 30 to 70 years undergoing TURBT for lateral and posterolateral wall bladder tumors who fulfilled the American Society of Anesthesiologists grade I and II criteria. The patients were divided into two groups: group U (n = 120) included patients who underwent ONB using an ultrasound-guided technique and group UN (n = 120) included patients who underwent ONB using ultrasound with the nerve stimulation technique. Block performance time, adductor jerks/spasms, adductor muscle power, and patient and surgeon satisfaction were compared. A P-value <0.05 was considered statistically significant.

Results

The mean block performance time in group U was significantly less (4.4 ± 0.82 minutes) than in group UN (6.55 ± 0.37 minutes). Compared to group U, group UN had significantly fewer adductor jerks/spasms during the surgery (7.76% vs. 20.35%, p = 0.006), significantly more surgeon satisfaction (92.24% vs. 79.65%, p = 0.006), significantly more patient satisfaction (92.24% vs. 79.65%, p = 0.006), and comparable complications (excessive bleeding and minor bladder injury) and adductor muscle power after the block (p > 0.05).

Conclusions

ONB using the nerve stimulation technique under ultrasound guidance has a longer mean block performance time, a higher success rate, and higher surgeon satisfaction than ONB under ultrasound guidance only.

## Introduction

Transurethral resection of bladder tumors (TURBT) is a routinely performed surgery for non-muscle invasive bladder tumors. This surgery can be performed under regional anesthesia (spinal anesthesia) or general anesthesia.

Regional anesthesia has many advantages such as lack of airway instrumentation, avoidance of polypharmacy, especially opioids, early recognition of bladder perforation, and fewer incidences of nausea and vomiting. The disadvantage of spinal anesthesia is the inability to prevent adductor spasms due to the obturator reflex [1].

The obturator reflex occurs when the obturator nerve is directly stimulated by the electrical current transmitted by the resectoscope, especially when the tumor is located on the lateral and posterolateral wall of the bladder, resulting in adductor spasm. This spasm is undesirable as it may result in disastrous consequences such as bladder perforation, excessive bleeding, and incomplete resection of tumors [1,2]. The frequency of severe adductor muscle jerks in patients undergoing TURBT for laterally located bladder tumors or large intraurethral prostatic adenomas is about 20-40% [3].

Apart from muscle relaxation with general anesthesia, several mechanisms to prevent the obturator reflex have been described in the literature. The use of laser resectors, reducing the intensity of the current of the resectoscope, change in the site of the inactive electrode, use of saline irrigation, superficial resection with low current and cutting with bipolar resectoscope, periprostatic infiltrations, and obturator nerve block (ONB) have been used with varying success to prevent adductor jerks [2-5]. Reducing the electrocoagulation voltage, incomplete bladder filling, or resection of thinner slices is not effective and may lead to incomplete resection of bladder tumors. Although bipolar systems and lasers have lowered adductor contraction, these modalities are expensive and unavailable in many centers. Therefore, spinal anesthesia with selective ONB is considered an appropriate option for elderly patients [6].

Obturator nerve blockade can be performed using the landmark approach described by Labat, ultrasound-guided and peripheral nerve stimulator-guided [6]. Ultrasonography and nerve stimulation modalities increase procedure efficacy by determining the exact site of the obturator nerve [7-9]. However, it is not known whether combining these two modalities would increase the success rate of ONB.

Therefore, this study was designed to compare the ultrasound-guided ONB and ultrasound with nerve stimulation-guided ONB for the prevention of adductor spasms in patients undergoing TURBT for lateral and posterolateral bladder wall tumors.

## Materials and methods

This randomized controlled study was conducted for 18 months in the Department of Anaesthesia & Intensive Care after obtaining approval from the hospital ethics committee (IEC/VMMC/SJH/THESIS/OCTOBER/2018-96). Written informed consent was obtained from all patients. The primary objective was to compare the success rate of ultrasound versus ultrasound with nerve stimulation-guided ONB to prevent adductor spasm in patients undergoing TURBT, as determined by adductor muscle power after the block, absence of adductor jerks during the surgery, and surgeon satisfaction. The secondary objectives were to compare the block performance time, number of needle passes, and patient satisfaction.

Adult patients aged 30 to 70 years undergoing TURBT for lateral and posterolateral wall bladder tumors who fulfilled the American Society of Anesthesiologists (ASA) grade I and II criteria were included in the study. Patients with pre-existing obturator nerve injury, adductor muscle weakness, local anesthetic allergy, infection at the site of injection, abnormal coagulation studies, and those who refused to provide consent were excluded. The study was conducted among 240 adult patients undergoing elective surgery under spinal anesthesia. Patients were randomized into the following two groups: group U (n = 120) included patients who underwent ONB using an ultrasound-guided technique, and group UN (n = 120) included patients who underwent ONB using ultrasound with a nerve stimulation technique.

Randomization was done using a sealed envelope system. Ten randomly generated treatment allocations within sealed opaque envelopes assigning U and UN in five envelopes each were prepared. After obtaining proper consent from the patient, an envelope was opened and the patient was offered the allocated group (patients were randomized in a series of blocks of 10). This was a single-blinded study.

Sample size

The sample size was calculated based on the study of Shah et al. [8], who reported a success rate of 76.7% using ultrasound-guided ONB and 90% using ultrasound with nerve stimulation. Taking these values as a reference, the minimum required sample size with an 80% power of study and a 5% level of significance was 120 patients in each study group. Hence, the total sample size was 240.

Preoperative preparation

Pre-anesthetic checkup of the patients was done. All patients were made to fast overnight and received a tablet of alprazolam 0.25 mg, a tablet of ranitidine 150 mg, and a tablet of metoclopramide 10 mg orally the night before surgery and two hours before surgery.

Anesthesia technique

After the pre-anesthetic checkup, patients were shifted to the operating room. Standard monitors for non-invasive blood pressure, electrocardiography, and pulse oximetry were attached. After securing intravenous access, intravenous fentanyl 50 µg was administered.

ONB was given to the patients in a supine position with the leg flexed at the knee joint and the thigh slightly abducted and externally rotated. An ultrasound machine, Kontron Medical (L5- 10S5), with a high frequency (5-10 MHz) linear array ultrasound transducer probe was used to administer the blocks. All blocks were performed by the same anesthesiologist in both groups.

Ultrasound-guided technique

A high-frequency linear probe was placed transversally at the inguinal crease to identify the femoral artery and vein. The transducer was traced medially to identify a beak-shaped muscle, pectineus. Further medially, adductor longus, adductor brevis, and adductor magnus muscles were identified. The most anterior was the adductor longus, the next posterior was the adductor brevis, and the most posterior and largest was the adductor magnus. Under all aseptic precautions, a skin wheal was raised with 1 mL of 2% isobaric lignocaine, and a 21-gauge 100 mm SonoPlex needle was introduced in the plane with the transducer. The needle was advanced from the lateral side of the probe toward the medial direction. In group U, the needle was advanced medially in the fascial plane between the adductor longus and the adductor brevis. After negative aspiration and confirming the location of the needle tip with hydrodissection using 2 mL of normal saline, 5 mL of 0.5% isobaric bupivacaine was injected in the interfascial plane to block the anterior division of the obturator nerve. The needle was withdrawn to the skin and redirected medially toward the fascial plane between the adductor brevis and adductor magnus. After negative aspiration and confirmation with hydrodissection, 5 mL of 0.5% isobaric bupivacaine was deposited to block the posterior division of the obturator nerve.

Ultrasound with the nerve stimulator-guided technique

In group UN, a 21-gauge 100 mm Stimuplex insulated needle attached to a nerve stimulator was advanced as described above. As the needle tip was advanced between the fascial plane of the adductor longus and brevis, on stimulation of the anterior division of the obturator nerve adductor, twitches were noticed at a current of 1.5 mA. When adductor twitches were present at less than or equal to 0.5 mA, 5 mL of 0.5% isobaric bupivacaine was injected in the interfascial plane. Similarly, for the posterior division, when adductor twitches were present at less than or equal to 0.5 mA, the drug was deposited in the interfascial plane between the adductor brevis and adductor magnus.

The number of needle passes was noted and counted as the number of times the needle was withdrawn and redirected. Block performance time was noted as the time taken from the start of ultrasonography to needle removal.

Adductor muscle power was assessed after 15 minutes using the following grading [10]: 0, no observable adductor muscle contraction; 1, unable to adduct the hip joint with gravity elimination; 2, able to adduct the hip joint through a full range of motion with gravity elimination; 3, able to adduct the hip joint against gravity; 4, able to adduct the hip joint against moderate resistance; and 5, able to adduct the hip joint against maximal resistance. Grade 2 or less was considered a successful block. Patients who had adductor muscle power of a grade more than 2 were excluded from further study.

After the assessment of the block, a subarachnoid block was administered under all aseptic precautions at the L4-L5 intervertebral space in the lateral position. For this, 2.2 mL of 0.5% hyperbaric bupivacaine with 10 µg fentanyl was administered intrathecally. After confirming the sensory blockade at the T-T10 level, surgery was started. Patients were continuously monitored throughout the surgery.

The success rate of the block was determined by the absence of adductor jerks/spasms during the surgery. Intraoperatively, if any jerks were observed which led to the discontinuation of surgery, the surgeon was asked to decrease the current of the electrocautery. If the adductor jerks continued, general anesthesia with muscle relaxant was given, and it was considered a failure of technique. Complications such as bleeding, bladder injury, and bladder perforation were noted. At the end of the surgery, the satisfaction of the surgeon and patient was noted.

Statistical analysis

Categorical variables were presented as numbers and percentages (%) and continuous variables as mean ± SD and median. The normality of data was tested using the Kolmogorov-Smirnov test. If the normality was rejected, a non-parametric test was used. Quantitative variables were compared using the independent t-test or the Mann-Whitney test (when the data sets were not normally distributed) between the two groups. Qualitative variables were compared using the chi-square test or Fisher’s exact test. A p-value <0.05 was considered statistically significant. The data were entered in an MS Excel spreadsheet (Microsoft Corp., Redmond, WA, USA), and analysis was done using SPSS version 21.0 (IBM Corp., Armonk, NY, USA).

## Results

**Figure 1:**
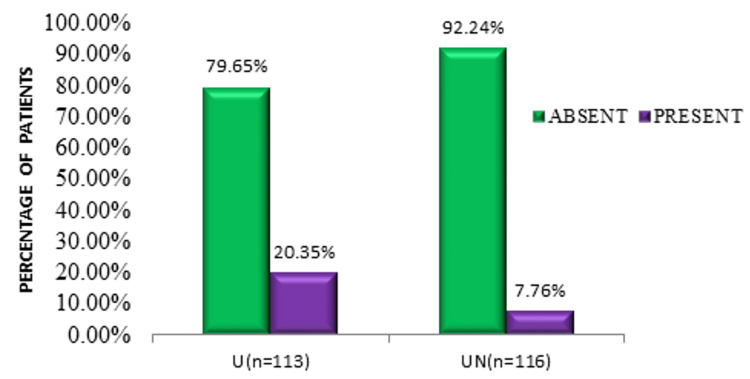
Comparison of adductor jerks/spasms during surgery between groups U and UN.

**Figure 2:**
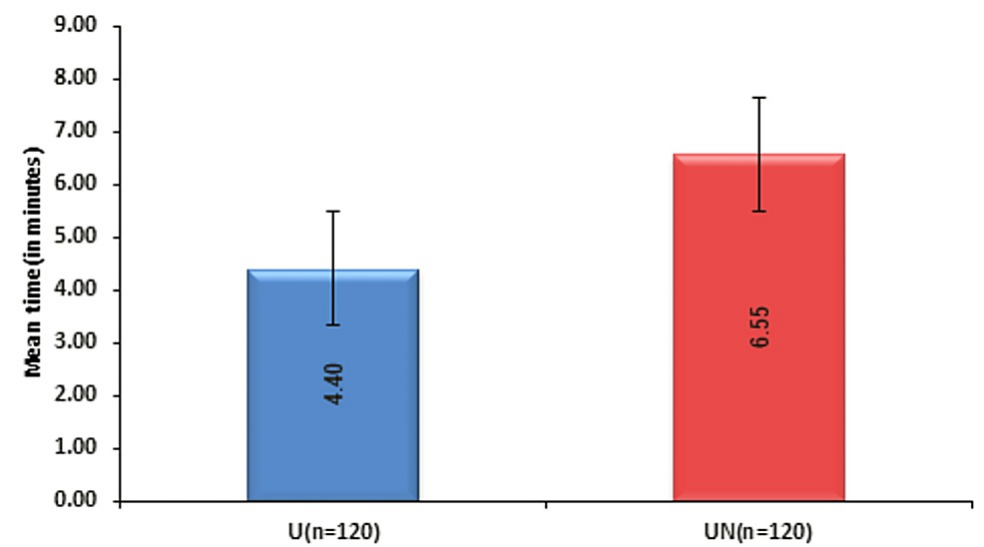
Comparison of block performance time (in minutes) between groups U and UN.

Both groups were comparable in terms of patient profile and ASA status (Table 1). Post-block adductor muscle weakness was comparable in both groups. Seven patients in group U and four patients in group UN had adductor muscle power of a grade more than 2 and were excluded from the study. Group UN had significantly fewer adductor jerks/spasms intraoperatively compared to group U (7.76% (n = 116) vs. 20.35% (n = 113), p = 0.006) (Figure 1). Surgeon satisfaction (92.24% vs. 79.65%, p = 0.006) and patient satisfaction (92.24% vs. 79.65%, p = 0.006) was significantly better in group UN (n = 116) than in group U (n = 113). A statistically significant difference was seen in the block performance time (in minutes) between groups U and UN (p < 0.05) (Figure 2). The mean number of needle passes and complications were comparable in both groups (Table 2). No significant difference was seen in the distribution of complications (excessive bleeding and minor bladder injury) between groups U and UN (p = 0.211).

**Table 1:** Comparison of patient profile between groups U and UN. Data are represented as n, and p-values <0.05 are considered significant.

Sociodemographic characteristics	Group U (n = 120)	Group UN (n = 120)	P-value
Age (years), mean ± SD	56.95 ± 5.1	57.28 ± 4.67	0.607
Gender			
Female (%)	19 (15.83%)	15(12.50%)	0.459
Male (%)	101 (84.17%)	105 (87.50%)	
Height (cm), mean ± SD	163.21 ± 3.08	162.86 ± 2.08	0.389
Weight (kg), mean ± SD	64.8 ± 2.95	64.32 ± 2.43	0.174
American Society of Anesthesiologists grade			
1 (%)	88 (73.33%)	98 (81.67%)	0.122
2 (%)	32 (26.67%)	22 (18.33%)	

**Table 2:** Comparison of outcomes between groups U and UN. Data are represented as n and percentage. P-values <0.05 are considered significant. Seven patients in group U and four patients in group UN had adductor muscle power of a grade of more than 2 and were excluded from further study.

Outcome (%)	Group U (n = 120)	Group UN (n = 120)	P-value
Adductor muscle power after the block			
Grade 1	30 (25%)	40 (33.33%)	0.215
Grade 2	83 (69.17%)	76 (63.33%)	
Grade 3	3 (2.50%)	0 (0%)	
Grade 4	4 (3.33%)	4 (3.33%)	
Outcome (%)	Group U (n = 113)	Group UN (n = 116)	P-value
Adductor jerks/spasms during the surgery (yes)	23 (20.35%)	9 (7.76%)	0.006
Surgeon satisfaction (yes)	90 (79.65%)	107 (92.24%)	0.006
Patient satisfaction (yes)	90 (79.65%)	107 (92.24%)	0.006
Complications (excessive bleeding and minor bladder injury)	7 (6.19%)	3 (2.59%)	0.211
Excessive bleeding	5 (4.42%)	2 (1.72%)	
Minor bladder injury	2 (1.76%)	1 (0.86%)	

## Discussion

**Figure 3:**
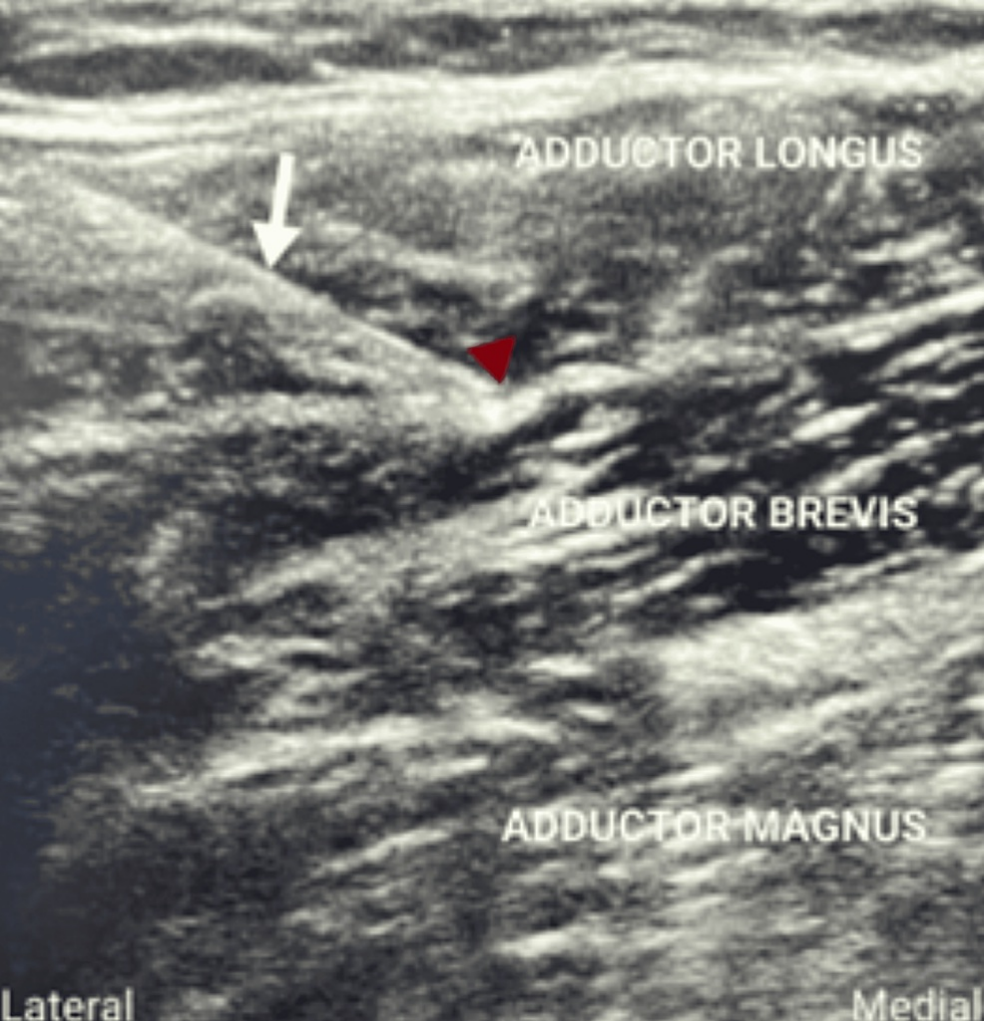
The white arrow shows the needle and the red arrowhead shows the needle tip between the adductor longus and adductor brevis to block the anterior branch of the obturator nerve.

**Figure 4:**
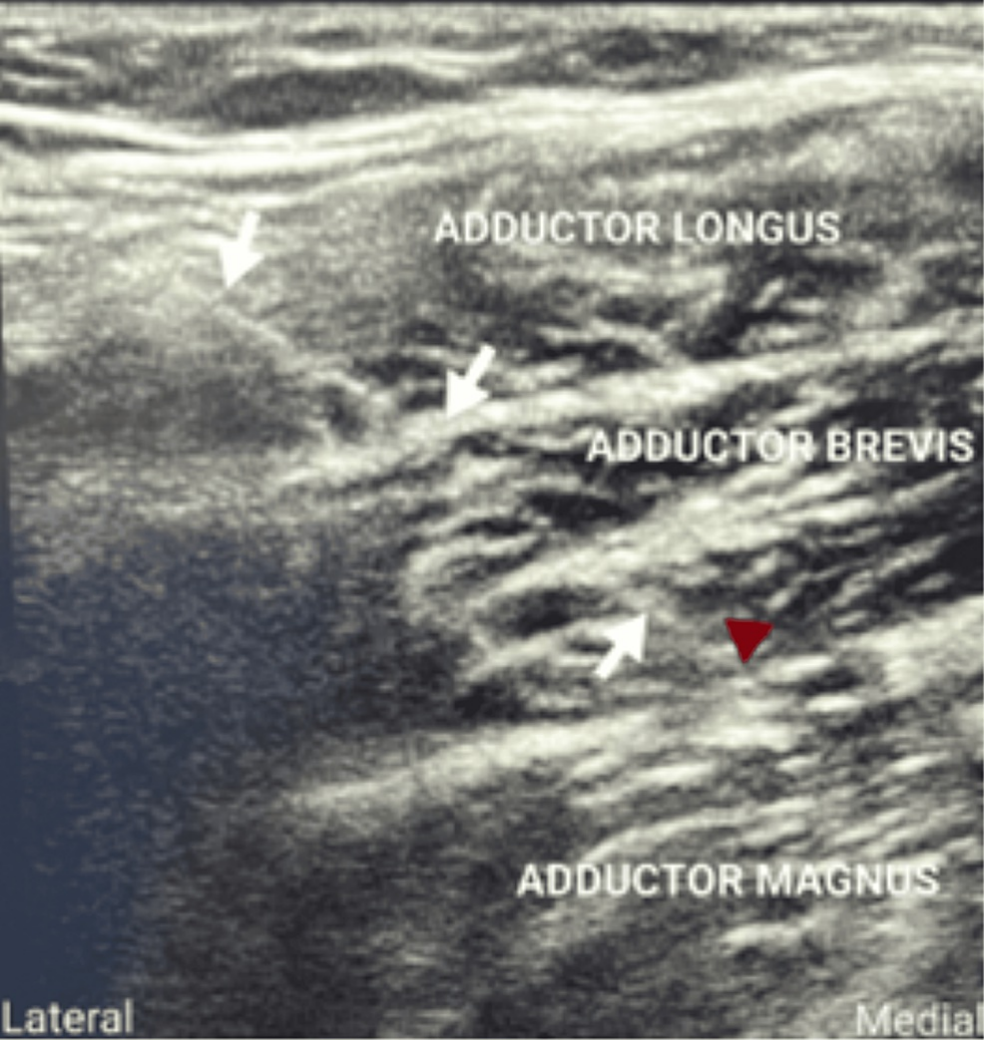
White arrows show the needle and the red arrowhead shows the needle tip between the adductor brevis and adductor magnus to block the posterior branch of the obturator nerve.

The obturator nerve arises from the anterior rami of the second, third, and fourth lumbar nerves; descends through psoas major and emerges from its medial border; and then runs along the lateral wall of the lesser pelvis and extends to the anterior thigh. During its course, it divides into anterior and posterior branches as it emerges from the obturator canal, separated by some fibers of the obturator externus muscle. The anterior obturator nerve branch initially passes through the interfascial plane between the pectineus and adductor brevis muscles. Further, it runs between the adductor longus and adductor brevis muscles (Figure 3), innervating the adductor longus and brevis and gracillus. The posterior branch passes between the fascial planes of the adductor brevis and adductor magnus muscle (Figure 4).

Ultrasound-guided ONB techniques are classified into distal or proximal. In the distal approach, the anterior and posterior branches of the obturator nerve are blocked separately, while in the proximal technique, the obturator nerve is blocked at the level of the interfascial plane between the pectineus and obturator externus muscles, before it divides into anterior and posterior branches. The proximal approach is technically difficult and carries a greater risk of a vascular puncture, making it the less preferred approach [10,11].

Post-block muscle weakness and success rate

In this study, adductor muscle power was assessed after the block. Overall, 96% of patients in group UN and 94% of patients in group U had grade 1 or 2 adductor muscle weakness post-ONB (p = 0.215). However, the success rate, as determined by the absence of adductor jerks intraoperatively, was 92.24% in group UN which was significantly higher compared to 79.65% in group U (p = 0.006). Despite effectively blocking the obturator nerve, undesirable adductor muscle spasms may still occur during TURBT even when an obturator nerve block is performed correctly due to variations in the obturator nerve’s ramifications [11].

The efficacy rate of ultrasound-guided ONB is mentioned as 84% and 96%, as per the literature [12]. In a clinical report by Lee et al. [13], ONB was given under ultrasound guidance to 25 patients. Adductor muscle strength was measured after the block which was decreased in all patients. The adductor jerks were successfully inhibited in 96% of the cases. In our study, adductor jerks were inhibited in 79.65% of the patients in whom ONB was given under ultrasound guidance. The lower success rate in our study could be attributed to the larger sample size of our study. Shah et al. [8] compared ONB under ultrasound visualization (group I) and ONB under ultrasound guidance with the nerve stimulation technique (group II). They observed a success rate of 90% in group II compared to 76.6% in group I (p = 0.299), which is comparable to our study. They did not elaborate on post-block adductor weakness. Manassero et al. [14] enrolled 50 patients undergoing TURBT and divided them into two groups. Group US received ONB under ultrasound guidance and group USENS received ONB under ultrasound guidance with the nerve stimulation technique. The success rate was 100% in the USENS group and 88% in the US group (p = 0.23) which is comparable to our study.

Mean block performance time

The mean block performance time in group UN was 6.55 ± 0.37 minutes which was longer than the mean block performance time of 4.4 ± 0.82 minutes in group U (p < 0.05). This can be attributed to the fact that nerve stimulation is a slightly prolonged procedure that checks for muscle twitches for both divisions in addition to ultrasound visualization. Although statistically significant, a delay of approximately 120 seconds may not be clinically significant, especially if it increases the efficacy of the procedure. Shah et al. [8] observed a mean block performance time of 4.47 ± 0.73 minutes in group II versus 2.10 ± 0.51 minutes in group I (p < 0.05) which parallels our study. Manassero et al. [14] also observed a longer performance time in the USENS than in the US group (3.0 vs. 1.6 minutes, p < 0.001) which is similar to our study. Yangtse et al. [15] compared ONB under ultrasound guidance and ONB using the nerve stimulation technique under ultrasound guidance. The mean block performance time was longer in the latter group (p < 0.05) which is similar to our study.

Number of needle passes

There was no statistically significant difference in the number of needle passes between the two groups. The mean number of needle passes in group U was 2.17 ± 0.42 and in group UN was 2.17 ± 0.46 (p = 1) which is in congruence with the study by Shah et al. [8].

Surgeon and patient satisfaction

Surgeon satisfaction was present in 92.24% of the patients in group UN which was significantly higher compared to 79.65% in group U (p = 0.006). Patient satisfaction was present in 92.24% of the patients in group UN which was significantly higher compared to 79.65% in group U (p = 0.006). Shah et al. [8] observed a surgeon satisfaction of 90% in group II and 76.7% in group I (p = 0.299) and patient satisfaction of 96.7% in group II and 86.7% in group I (p = 0.353). These results were parallel to the results of our study.

Complications

In this study, excessive bleeding and minor bladder injury were seen in seven (6.19%) patients in group U and three (2.59%) patients in group UN. No statistically significant difference was observed between the two groups (p = 0.211), but this result may be clinically important and implies that a more accurate and precise blockade is important even if the block performance time is longer. Literature shows the incidence of bladder perforation from 0.9% to 5% [16]. Dick et al. reported a 13% incidence of hemorrhage [17].

Limitations of the study

This study has a few limitations. First, this was a single-center study. Second, there is a tedious learning curve in recognizing the various structures using ultrasound (sonoanatomy) and guiding the needle to the appropriate target.

## Conclusions

Our findings show that an ONB along with spinal anesthesia is a safe and effective method to prevent adductor jerks/spasms in patients undergoing TURBT for lateral and posterolateral bladder wall tumors. ONB using the nerve stimulation technique under ultrasound guidance has a longer mean block performance time, a higher success rate, and higher surgeon satisfaction than ONB under ultrasound guidance only.
